# Coronary Dominance on Coronary CT Angiography: Prevalence and Potential Implications for Electrocardiographic Correlation

**DOI:** 10.3390/jcm15135258

**Published:** 2026-07-06

**Authors:** William Andrés Prada Mancilla, Katherine Díaz, Fernando Ortiz

**Affiliations:** Department of Cardiac Imaging, Clínica Reina Sofía, Bogota 110121, Colombia

**Keywords:** coronary dominance, tomography, myocardial infarction

## Abstract

**Background/Objectives:** Coronary dominance is a key anatomical variant in the interpretation of coronary artery disease and its correlation with electrocardiographic (ECG) findings. While right dominance is the most common pattern, left dominance may influence clinical and ECG presentations. This study aimed to determine the prevalence of coronary dominance using coronary CT angiography (CCTA) and evaluate its association with disease severity, gender, and ECG findings. **Methods:** A retrospective observational analytical study was conducted including 677 patients who underwent CCTA. Coronary dominance was classified as right, left, or codominant. Disease severity was assessed using CAD-RADS. Demographic, clinical, anatomical, and ECG variables were analyzed. Statistical analysis included descriptive statistics, chi-square tests, and multivariable logistic regression analysis. **Results:** Right coronary dominance was predominant (89.7%), followed by left dominance (8%) and codominance (2.3%). No significant association was found between coronary dominance and disease severity or gender (*p* = 0.32). Male gender was significantly associated with severe disease (CAD-RADS 4–5) (*p* < 0.005). The left anterior descending (LAD) artery was the most frequently affected vessel. In the left dominance subgroup, 72.2% of patients had a normal ECG, with a low prevalence of ischemic findings (3%). Cases of left dominance with severe disease and confirmed acute coronary syndrome were identified. **Conclusions:** Coronary dominance is not associated with disease severity or gender; however, left dominance represents a clinically relevant finding due to its association with atypical or normal ECG presentations. Its systematic inclusion in CCTA reports is recommended to improve clinical correlation and decision-making.

## 1. Introduction

Coronary artery disease (CAD) remains one of the leading causes of morbidity and mortality worldwide, driving the increasing use of non-invasive cardiovascular imaging techniques such as coronary computed tomography angiography (CCTA) [1]. CCTA enables detailed anatomical assessment of the coronary arteries with high diagnostic accuracy and has become an established tool in the evaluation of suspected CAD, particularly because of its high negative predictive value and its ability to characterize atherosclerotic disease using standardized reporting systems such as CAD-RADS (Coronary Artery Disease—Reporting and Data System) [1,2].

**Figure 1:**
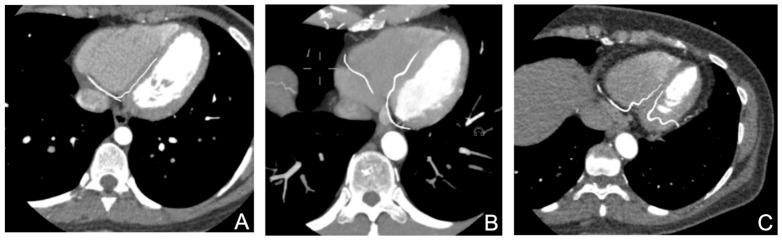
Coronary dominance on CCTA. (**A**) Right coronary dominance. (**B**) Left coronary dominance. (**C**) Codominance. Images were obtained utilizing maximum intensity projection (MIP) reconstructions on a 160-slice CT scanner at Clínica Reina Sofía.

Beyond the assessment of coronary stenosis, CCTA provides comprehensive anatomical characterization of the coronary circulation, including the evaluation of coronary dominance. Coronary dominance is defined by the arterial origin of the posterior descending artery and determines the vascular supply to the inferior and posterior myocardial territories [3]. Right coronary dominance is the most common anatomical pattern and serves as the basis for most conventional electrocardiographic (ECG) interpretation models in ischemic heart disease [4]. However, a proportion of patients present with left coronary dominance or codominance (Figure 1), anatomical variants that may influence the relationship between coronary anatomy, myocardial ischemia distribution, and ECG findings, particularly in cases involving left circumflex artery (LCX) disease [3,4].

Although coronary dominance is routinely identifiable on CCTA, it is inconsistently incorporated into structured imaging reports and clinical interpretation. Most prior studies have focused primarily on its anatomical prevalence or prognostic implications, while its integration into ECG–clinical correlation remains less explored [5,6,7]. This may be particularly relevant in patients with LCX-related ischemia, in whom standard 12-lead ECG findings may be subtle, atypical, or potentially non-diagnostic, especially in the setting of left coronary dominance [8].

In contemporary cardiovascular imaging, CCTA interpretation increasingly extends beyond the identification of anatomical stenosis and incorporates structured reporting systems, plaque characterization, and emerging functional assessment strategies. Within this context, systematic recognition of coronary dominance may contribute to improved anatomical–clinical integration and support interpretation in selected patients with possible ECG-imaging discordance.

Therefore, the aim of this study was to describe the prevalence of coronary dominance patterns in a cohort of patients undergoing CCTA and to explore their relationship with CAD severity, anatomical distribution of coronary lesions, and ECG findings in a real-world clinical population.

## 2. Methodology

### 2.1. Study Design and Population

A retrospective observational analytical study was conducted based on consecutive patients who underwent clinically indicated CCTA between January 2024 and January 2026 at a tertiary cardiovascular imaging center. The primary objectives were to determine the prevalence of coronary dominance patterns and to evaluate their relationship with coronary artery disease severity, anatomical distribution of lesions, and ECG findings.

A total of 755 patients were initially screened. Among them, 71 patients underwent only non-contrast coronary calcium scoring without contrast-enhanced CCTA and were therefore excluded from the coronary dominance analysis, since coronary dominance cannot be reliably determined without visualization of the posterior descending artery. Additionally, 7 patients were excluded due to non-diagnostic image quality or incomplete clinical/electrocardiographic information. The final analytical cohort included 677 patients with diagnostic-quality CCTA studies.

A STROBE-based patient selection flowchart was constructed to describe the screening, exclusion, and inclusion process.

### 2.2. Inclusion and Exclusion Criteria

Patients aged ≥ 18 years who underwent clinically indicated CCTA for evaluation of suspected or known coronary artery disease were included.

Exclusion criteria were:

- Non-diagnostic CCTA studies due to severe motion artifacts or extensive coronary calcification impairing coronary evaluation;
- Incomplete demographic, clinical, or electrocardiographic data;
- Absence of adequate anonymization of imaging and clinical records;
- Patients who underwent calcium scoring alone without contrast-enhanced coronary angiography.

Because this was a retrospective anonymized study, the institutional ethics committee waived the requirement for individual informed consent according to institutional regulations and national ethical standards.

### 2.3. CCTA Acquisition Protocol

All examinations were performed using a 160-slice multidetector CT scanner (Canon Aquilion Lightning, Otawara, Japan) according to standardized institutional cardiovascular imaging protocols.

All patients initially underwent non-contrast coronary artery calcium scoring for quantification of coronary calcification burden using the Agatston method [6].

Beta-blockers and sublingual nitrates were administered when clinically appropriate to optimize heart rate control and coronary vasodilation.

Subsequently, contrast-enhanced CCTA was performed using ECG-gated acquisition, either prospective or retrospective depending on heart rate and rhythm characteristics.

Acquisition parameters included:

- Tube voltage: 100–120 kVp;
- Automated tube current modulation;
- Intravenous administration of weight-adapted iodinated contrast (60–80 mL) at a flow rate of 4.5–6.0 mL/s (injection duration: 12–15 s), followed by a saline flush;
- Slice thickness reconstruction between 0.5–0.75 mm.

Multiphase image reconstruction covering 10–90% of the R–R interval was routinely performed. The 75% diastolic phase was systematically used as the primary phase for coronary artery analysis due to lower motion artifact susceptibility, with additional phases reviewed when necessary.

Post-processing included multiplanar reformations (MPR), curved planar reformations, maximum intensity projections (MIP), and three-dimensional angiographic reconstructions. Post-processing analysis of all images was performed using the Vitrea (version 7.16) advanced visualization system.

### 2.4. Image Analysis and Definitions

All CCTA studies were independently reviewed by radiologists specialized in cardiovascular imaging with more than five years of experience. Image reviewers were blinded to clinical outcomes and, when available, to invasive angiographic findings performed due to clinical indications.

Coronary dominance was classified according to the origin of the posterior descending artery (PDA):

- Right dominance: PDA originating from the right coronary artery (RCA);
- Left dominance: PDA originating from the left circumflex artery (LCX);
- Codominance: Contribution from both RCA and LCX.

Coronary artery disease severity was assessed using the CAD-RADS classification system (version 2.0), categorizing disease from CAD-RADS 0 to CAD-RADS 5 according to maximal coronary stenosis severity. Coronary calcium burden was additionally evaluated using Agatston calcium score categories.

The anatomical distribution of coronary lesions was recorded for:

- Left anterior descending artery (LAD);
- Left circumflex artery (LCX);
- Right coronary artery (RCA);
- Posterior descending artery (PDA).

Significant coronary artery disease was defined as CAD-RADS 4–5.

### 2.5. Electrocardiographic and Clinical Data

Clinical and demographic variables were retrospectively obtained from institutional electronic medical records and included:

- Age;
- Sex;
- Hypertension;
- Diabetes mellitus;
- Dyslipidemia;
- Smoking history;
- Clinical indication for CCTA.

Electrocardiograms (ECGs) performed within the same clinical episode or closest temporal interval to the CCTA study were reviewed from medical records. ECG interpretation was based on the formal cardiology report available in the institutional system.

ECG findings were categorized into:

- Normal ECG;
- Ischemic changes;
- Repolarization abnormalities;
- Tachyarrhythmias;
- Bradyarrhythmias;
- Mixed tachy-brady arrhythmias.

The ECG analysis was primarily focused on patients with left coronary dominance due to the study hypothesis regarding potential ECG–clinical discordance in this subgroup. Consequently, ECG findings were not systematically coded in all patients with right coronary dominance, particularly in cases with normal ECGs, resulting in missing ECG data within the overall cohort.

No patients included in the study underwent systematic posterior or extended ECG lead acquisition (V7–V9), limiting the assessment of posterior ischemia related to left circumflex artery involvement.

Because of these limitations, ECG analyses should be interpreted as exploratory and hypothesis-generating rather than definitive population-level associations.

### 2.6. Statistical Analysis

Statistical analysis was performed using Jamovi software (version 2.7.26).

Continuous variables were expressed as mean ± standard deviation or median with interquartile range (IQR) depending on distribution normality, which was assessed using the Shapiro–Wilk test. Categorical variables were expressed as frequencies and percentages.

A univariate descriptive analysis was initially performed to characterize demographic, anatomical, clinical, and electrocardiographic variables.

Bivariate analyses were subsequently conducted using:

- Chi-square (χ^2^) test for categorical variables;
- Fisher’s exact test when expected cell frequencies were <5.

Associations evaluated included:

- Coronary dominance and sex;
- Coronary dominance and CAD-RADS severity;
- Coronary dominance and ECG findings;
- Coronary dominance and anatomical lesion distribution;
- Sex and CAD severity.

Effect sizes were reported using odds ratios (OR) with 95% confidence intervals (95% CI).

To strengthen the analytical model and adjust for potential confounding variables, a multivariable logistic regression analysis was additionally performed.

The multivariable model evaluated predictors of severe coronary artery disease, defined as CAD-RADS 4–5.

Dependent variable: Severe coronary artery disease (CAD-RADS 4–5).

Independent variables: Age, sex, hypertension, diabetes mellitus, dyslipidemia, smoking history, and coronary dominance pattern.

Adjusted odds ratios (aOR) with 95% confidence intervals were calculated for the multivariable model.

A two-sided *p*-value < 0.05 was considered statistically significant. Due to the exploratory nature of subgroup analyses involving left-dominant circulation, findings were interpreted cautiously.

### 2.7. Ethical Considerations

The study was conducted in accordance with the Declaration of Helsinki, institutional ethical standards, and Colombian Resolution 8430 of 1993. The institutional ethics committee approved the study protocol and classified the project as minimal-risk retrospective research. All patient data were anonymized prior to analysis, and no direct patient intervention was performed.

## 3. Results

**Figure 2:**
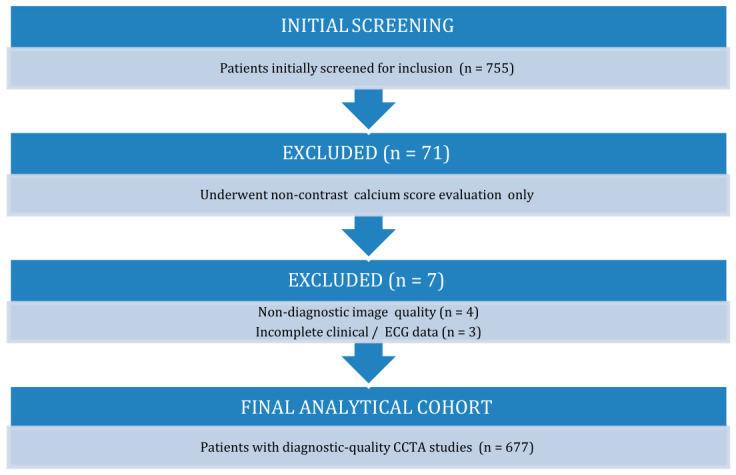
STROBE flowchart.

A total of 755 patients were initially screened for inclusion. Seventy-one patients who underwent non-contrast calcium score evaluation only were excluded from the coronary dominance analysis. Additionally, 7 patients were excluded because of non-diagnostic image quality or incomplete clinical/electrocardiographic data. The final analytical cohort therefore included 677 patients with diagnostic-quality CCTA studies available for coronary dominance assessment and CAD-RADS classification (Figure 2).

The mean age of the study population was 59.7 ± 12.6 years, with a median age of 60 years (range: 21–94 years). According to the Shapiro–Wilk test, age distribution was non-normal (*p* = 0.001).

Right coronary dominance was the predominant anatomical pattern, identified in 607 patients (89.7%), followed by left coronary dominance in 54 patients (8.0%) and codominance in 16 patients (2.3%) (Table 1).

When coronary dominance was analyzed according to CAD-RADS severity categories, right dominance remained the predominant pattern across all CAD-RADS groups. No statistically significant association was identified between coronary dominance pattern and CAD severity.

Regarding sex distribution, 326 patients (48.1%) were female and 351 (51.9%) were male. Right coronary dominance was the predominant pattern in both sexes, with no statistically significant association between sex and coronary dominance pattern (χ^2^ test, *p* = 0.32) (Table 2).

In the analysis of CAD severity according to sex, no statistically significant association was observed across the overall CAD-RADS distribution (*p* = 0.10). However, after grouping patients with severe coronary artery disease (CAD-RADS 4A, 4B, and 5), male sex was significantly associated with severe disease (*p* < 0.005).

The anatomical distribution of coronary lesions showed a predominance of left anterior descending artery (LAD) involvement in both sexes, without statistically significant sex-related differences in lesion distribution (*p* = 0.34) (Table 3). A substantial proportion of patients presented multivessel coronary involvement; therefore, the frequencies shown in Table 3 correspond to the predominant or most clinically significant arterial territory identified on CCTA and should not be interpreted as mutually exclusive categories summing to the total study population.

A multivariable logistic regression model was subsequently performed to identify independent predictors of severe coronary artery disease (CAD-RADS 4–5). Variables included in the model were age, sex, hypertension, diabetes mellitus, dyslipidemia, smoking history, and coronary dominance pattern.

Increasing age (adjusted OR 1.06; 95% CI: 1.03–1.09; *p* < 0.001) and male sex (adjusted OR 5.89; 95% CI: 2.53–13.66; *p* < 0.001) were independently associated with severe CAD. In contrast, left coronary dominance was not independently associated with severe coronary artery disease after multivariable adjustment (adjusted OR 0.95; 95% CI: 0.28–3.29; *p* = 0.945) (Table 4).

Analysis of cardiovascular risk factors showed no statistically significant differences between sexes (χ^2^ = 16.0; *p* = 0.315). Dyslipidemia was the most frequent cardiovascular risk factor, followed by the presence of multiple combined risk factors.

ECG analysis was primarily focused on the subgroup of patients with left coronary dominance due to the exploratory nature of the study hypothesis regarding potential ECG–clinical discordance in this anatomical variant. Among patients with left dominance, 72.2% presented with a normal ECG, while the remaining patients showed repolarization abnormalities (5.6%), tachyarrhythmias (5.6%), bradyarrhythmias (11.1%), combined tachy-brady arrhythmias (1.9%), and ischemic findings in 3.7% of cases (Table 5).

No patients included in the study underwent systematic extended posterior ECG lead acquisition (V7–V9).

**Figure 3:**
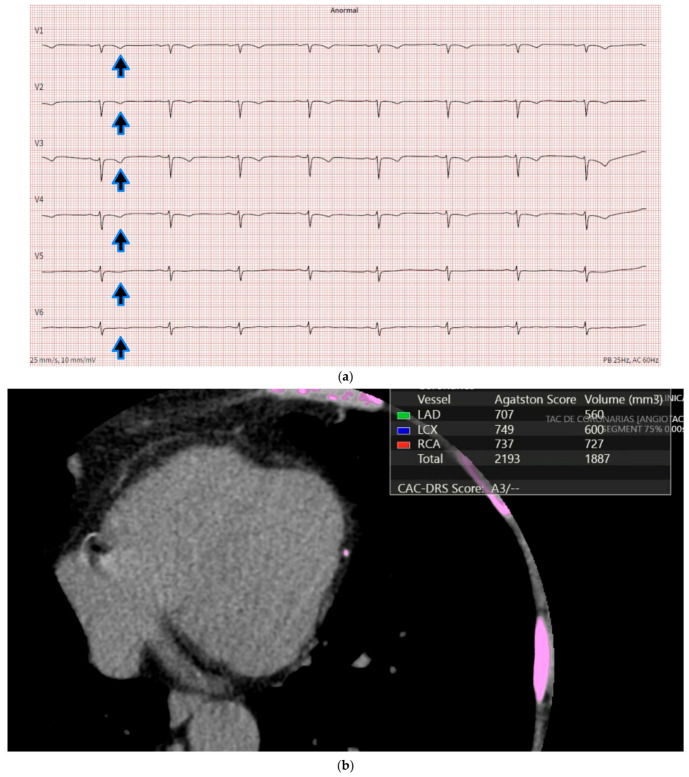

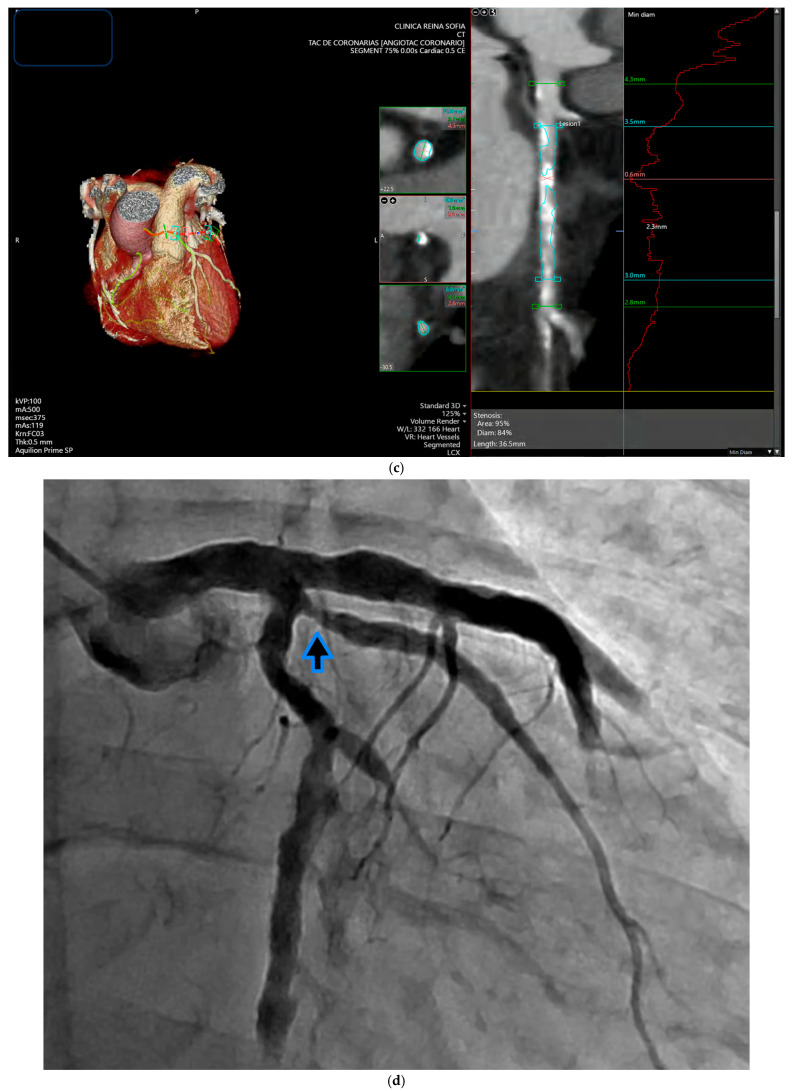
A 61-year-old male with hypertension and dyslipidemia, presenting with ACS and left coronary dominance. (**a**) ECG showing subepicardial ischemia in the anterior and lateral walls (arrow). (**b**) Calcium score box shows evidence of high atherosclerotic plaque burden in the circumflex artery (LCX). Blue box with Agatston score of 749. The purple color is for segmentation selection. (**c**) Angiographic reconstruction of the LCX with stenosis grade CAD-RADS 4a. The red line in the 3D reconstruction represents the LCX. The green boxes represent the analysis limit, the aquamarine boxes the evaluated artery segment, and the red dot the point of greatest stenosis. Similarly, in the axial and longitudinal representations, (**d**) Coronary catheterization with evidence of obstruction of the LCX. The blue arrow shows the first tract of the circumflex artery with moderate obstruction.

Additionally, two illustrative cases of patients with left coronary dominance and severe circumflex artery disease (CAD-RADS 4A) demonstrated ecg findings suggestive of acute coronary syndrome with subsequent angiographic confirmation of significant left circumflex artery obstruction (Figure 3). These cases are presented as illustrative clinical examples rather than inferential cohort-level evidence.

## 4. Discussion

The present study evaluated the prevalence of coronary dominance patterns on CCTA and their relationship with CAD severity, anatomical lesion distribution, and ECG findings. Overall, the results are consistent with previously reported prevalence data while also highlighting clinically relevant considerations regarding the integration of coronary anatomy and ECG interpretation [5,6].

Right coronary dominance was the predominant anatomical pattern in our cohort, identified in 89.7% of patients, followed by left dominance (8.0%) and codominance (2.3%). These findings are consistent with prior angiographic and CCTA studies reporting right dominance as the most common coronary circulation pattern [7,8]. From a physiological perspective, this predominance implies that the inferior and posterior myocardial territories are supplied primarily by the right coronary artery in most patients, which forms the basis for the conventional electrocardiographic interpretation of inferior and posterior ischemia [9,10]. Nevertheless, approximately 10% of patients presented non-right dominant circulation patterns, emphasizing that relevant anatomical variability exists and may influence clinical and ECG interpretation [11,12,13].

No significant association was identified between coronary dominance pattern and coronary artery disease severity according to CAD-RADS classification. Right coronary dominance remained the most frequent pattern across all CAD-RADS categories, which likely reflects the baseline prevalence distribution within the cohort rather than a pathophysiological association. Furthermore, in the multivariable logistic regression model, left coronary dominance was not independently associated with severe coronary artery disease (CAD-RADS 4–5) after adjustment for age, sex, and cardiovascular risk factors. In contrast, increasing age and male sex were independently associated with severe CAD. These findings support the concept that coronary dominance represents an anatomical variant rather than an independent determinant of atherosclerotic disease burden [10,14].

Similarly, no statistically significant association was observed between sex and coronary dominance distribution (*p* = 0.32), suggesting that coronary dominance patterns are relatively independent of biological sex [10]. However, male sex demonstrated a significantly higher prevalence of severe CAD, both in unadjusted analysis and after multivariable adjustment, which is consistent with the established literature regarding sex-related differences in atherosclerotic disease burden and cardiovascular risk profiles [15].

Regarding anatomical lesion distribution, left anterior descending artery (LAD) involvement was the most frequent finding in both sexes, without statistically significant differences according to sex. A relevant proportion of patients presented multivessel disease, which explains why the anatomical categories shown in the analysis do not sum to the total study population. Although circumflex artery involvement was numerically more frequent in men, this finding did not reach statistical significance. Nevertheless, circumflex artery disease may have greater clinical relevance in patients with left coronary dominance because the left circumflex artery supplies a larger myocardial territory in this anatomical configuration [13,16].

An important aspect of this study is the exploratory ECG analysis in patients with left coronary dominance. Most patients in this subgroup presented with normal ECG findings (72.2%), while ischemic findings were identified in only a small proportion of cases. Although these findings should be interpreted cautiously given the limited sample size and exploratory design, they suggest that left coronary dominance alone does not necessarily produce abnormal ECG patterns in the absence of significant coronary disease [17,18]. However, when severe circumflex artery disease is present, ECG–clinical discordance may occur because ischemic territories supplied by the left circumflex artery are not always adequately represented on standard 12-lead ECGs [13].

Importantly, no patients in the present cohort underwent systematic acquisition of extended posterior ECG leads (V7–V9), which limited the evaluation of posterior ischemia related to circumflex artery involvement. This limitation is clinically relevant because posterior myocardial infarction and lateral ischemia secondary to left circumflex artery disease may be underrecognized on conventional ECG analysis [17].

The two illustrative cases included in this study demonstrated patients with left coronary dominance and severe circumflex artery stenosis (CAD-RADS 4A) who presented with ECG findings suggestive of acute coronary syndrome and subsequent angiographic confirmation of significant obstruction. These cases support the biological plausibility that coronary dominance may influence ECG–clinical correlation in selected scenarios. However, these examples should be interpreted as illustrative and hypothesis-generating rather than inferential evidence of a population-level association [12].

The analysis of cardiovascular risk factors showed no significant differences between sexes, with dyslipidemia representing the most prevalent risk factor in the cohort. This relative homogeneity suggests that the observed differences in severe CAD are likely multifactorial and influenced by additional biological and clinical variables beyond traditional risk factor prevalence alone [18,19].

Several limitations should be acknowledged. First, the retrospective design introduces potential selection and information bias. Second, ECG analysis was not systematically available for all patients, particularly those with right coronary dominance and normal findings, since the exploratory ECG analysis was primarily focused on patients with left dominance. Third, the relatively small number of patients with left dominance limited statistical power for subgroup analyses. Fourth, no systematic follow-up outcomes, invasive fractional flow reserve data, or major adverse cardiovascular event analysis were available; therefore, prognostic conclusions cannot be established from this cohort. Finally, the absence of posterior ECG lead acquisition limited the assessment of subtle ischemic presentations related to circumflex artery disease.

Overall, the present findings suggest that coronary dominance is not independently associated with coronary artery disease severity but remains clinically relevant for anatomical and electrocardiographic interpretation in selected patients, particularly those with left coronary dominance and significant circumflex artery disease [20]. These results reinforce the importance of integrating anatomical findings obtained from CCTA with clinical and ECG information to improve contextual interpretation and support individualized cardiovascular assessment.

## 5. Conclusions

In this cohort, right coronary dominance was the predominant anatomical pattern, while left dominance and codominance were less frequent variants. Coronary dominance was not independently associated with severe coronary artery disease after multivariable adjustment, whereas increasing age and male sex were significant predictors of severe CAD.

Although left coronary dominance was not associated with greater disease severity, its anatomical relevance remains clinically important in selected scenarios involving left circumflex artery disease, where standard electrocardiographic findings may be less characteristic or potentially non-diagnostic. The exploratory ECG findings observed in this study, together with the illustrative cases of severe LCX disease, support the concept that anatomical variants may influence the clinical–electrocardiographic correlation in specific patients.

These findings reinforce the importance of systematically reporting coronary dominance in CCTA studies as part of structured cardiovascular imaging interpretation. Integrating coronary dominance with CAD-RADS classification, plaque burden assessment, and clinical–electrocardiographic information may improve diagnostic interpretation and support more comprehensive clinical decision-making, particularly in patients with suspected ischemia and inconclusive standard ECG findings.

Further prospective multicenter studies with systematic ECG analysis, extended posterior lead evaluation, and longitudinal clinical outcomes are needed to better define the potential clinical and prognostic implications of coronary dominance patterns.

## Figures and Tables

**Table 1:** Prevalence of coronary dominance on CCTA.

Coronary Dominance	Frequency	%
Right dominance	607	89.7
Left dominance	54	8.0
Codominance	16	2.3

**Table 2:** Coronary dominance according to sex.

Sex	Right Dominance	Left Dominance	Codominance
Female	288	33	5
Male	319	21	11

**Table 3:** Anatomical distribution of coronary involvement by sex.

Sex	LAD	LCX	RCA	PDA	*p* Value
Female	82	9	10	0	0.34
Male	101	19	38	1	0.34

**Table 4:** Multivariable logistic regression model for severe coronary artery disease (CAD-RADS 4–5).

Variable	Adjusted OR	95% CI	*p*-Value
Age	1.06	1.03–1.09	<0.001
Male sex	5.89	2.53–13.66	<0.001
Left dominance	0.95	0.28–3.29	0.945

**Table 5:** ECG findings in patients with left coronary dominance.

ECG Finding	Frequency	%
Normal ECG	39	72.2
Repolarization abnormalities	3	5.6
Tachyarrhythmia	3	5.6
Bradyarrhythmia	6	11.1
Tachy-brady arrhythmia	1	1.9
Ischemic findings	2	3.7

## Data Availability

The original contributions presented in this study are included in the article. Further inquiries can be directed to the corresponding authors.

## Abbreviations

The following abbreviations are used in this manuscript:

ECG	Electrocardiographic
CCTA	Coronary CT angiography
LAD	Left anterior descending
LCX	Left circumflex
RCA	Right coronary artery
PDA	Posterior descending artery
CAD	Coronary artery disease
CAD-RADS	Coronary Artery Disease—Reporting and Data System
